# Old problems, new directions and upcoming requirements in participatory technology assessment

**DOI:** 10.1007/s10202-012-0116-3

**Published:** 2012-11-15

**Authors:** Michael Ornetzeder, Karen Kastenhofer

**Affiliations:** Vienna, Austria

Discussions on the role of participatory approaches in technology assessment and technology policy have a long history. While in the beginning this subject was handled mainly as a theoretical requirement for democratic governance of technology, active involvement of stakeholders and laypeople became popular in TA exercises throughout the 1980s. Since then, a variety of participatory TA (pTA) methods and strategies have been developed and widely used, raising further far-reaching expectations. It has been argued that participatory approaches might broaden and hence enrich the knowledge and value base in ongoing technological discourses and eventually improve the factual as well as democratic legitimacy of technology-related decisions (Joss and Bellucci 2002). Moreover, a stronger integration of diverse actors and stakeholders was linked to the promise of better socially embedded solutions, an increased acceptance and enhanced diffusion of technology as well as technology policy. However, practical experiences with pTA have shown that under real-world conditions, it is difficult to meet all these expectations (e.g. Abels and Bora 2004). Despite a continuing and widespread interest in pTA, empirical evidence and theoretical positions on the practical performance of pTA have remained ambiguous.

The papers selected for this special issue refer to this ambiguity from different angles and aim to contribute to the ongoing discussion on theoretical foundations, as well as practical experiences and critical appraisals of various forms of pTA. Most ideas, experiences and findings covered by this collection had first been presented and discussed at the yearly conference on technology assessment at the Austrian Academy of Sciences in 2011.^1^ In a similar vein, the papers in this special issue offer opportunities to sharpen our understanding of specific problems participatory approaches are confronted with seen from an insider’s point of view. At the same time, some contributions to this issue may stimulate discussion on the role and limitations of pTA in the light of ‘outside’ experiences, such as those against the backdrop of bottom-up civil engagement or participatory experiments in technology design. Taking a ‘more relaxed’ point of view may help redefine the role of pTA as one specific element in the wider context of technology governance. This does not mean that questions of legitimation or impact are of less importance in the future. They could, rather, open our eyes to new perspectives such as moving away from ‘purely’ participatory events to more comprehensive approaches, participation being one element among others. One of the case studies presented in this issue demonstrates that the role pTA is able to play within a specific political setting very much depends on the institutional arrangements and different national styles of policy-making. Other case studies, dealing with new procedural developments in the field, impressively show how practitioners of pTA try to react to upcoming requirements, overcome apparent problems and provide some valuable insights into the—sometimes puzzling—world of technology policy.

The first paper by Thomas Saretzki reminds us that it is of decisive importance to distinguish between technology assessment and technology policy when legitimation problems of participatory approaches are at stake. In contrast to technology policy, the core function of any modern TA is to mediate between three institutionally and functionally differentiated systems: science, politics and the public. According to Saretzki, legitimation problems indicate first of all that attempts to justify participation in a given case have not been entirely successful in the eyes of the relevant groups of sponsors, participants, organizers or observers. To deal with legitimation problems in a constructive way, Saretzki proposes the development of a multi-dimensional, self-reflective and self-critical approach to TA, which is able to serve as a system of reference for legitimating their own new roles, especially in the context of participatory procedures in TA.

Leo Hennen responds to recent criticism regarding practical experiments with pTA. According to this strand of literature, pTA shows a number of crucial problems. In many cases such public deliberation, processes have only marginal impact on political decisions. They also run the risk of being instrumentalized by influential interests groups while showing serious deficits regarding the production of new and authentic layperson expertise. In reference to these main lines of reasoning, Hennen argues in the paper that these criticisms insufficiently take into account the context of participatory TA as an element of policy consulting. Taking into account the specific nature of pTA as a strategy to stimulate public deliberation and collect attitudes, interests and patterns of argumentation used by laypersons, it is able to improve the responsiveness of the political system and to give a voice to perspectives that are not or only poorly represented in political debates and decision-making processes.

Against the background of civil society engagement in the fields of biomedicine and nanotechnology, Peter Wehling explores the potential of the so-called uninvited forms of participation and discusses possible consequences for more institutionalized formats of pTA. Similar to several other authors, Wehling refers to recently discussed practical problems and structural limitations with invited forms of pTA and contrasts these experiences with interest-based civil society interventions by patient associations and environmental and consumer organizations. He shows how uninvited initiatives in science and technology build up democratic legitimacy and manage to gain impact on decision-making processes. Wehling comes up with a number of recommendations to rethink and improve existing pTA approaches and methods and discusses new strategies to combine invited and uninvited forms of participation.

Based on two national case studies dealing with the governance of xenotransplantation in Switzerland and Austria, Erich Griessler explores the influence of structural conditions and national styles of policy-making on the role and effectiveness of pTA. Griessler shows that experiences with pTA differ fundamentally between the two countries. In Switzerland, the number of public dialogue exercises on xenotransplantation is much higher than in Austria and the possible impacts of these deliberations on policy-making seem to be much more effective. Griessler discusses a number of important similarities and differences regarding political institutions and practices of policy-making in both countries. He suggests that the most important factor for explaining the prominent role of pTA in Switzerland is the extraordinary veto power of the Swiss citizenry, which calls for dialogue formats to avoid potential resistance from the public.

Michael Decker and Torsten Fleischer report on recent experiences with, as they call it, ‘big style’ participation in Germany. Both authors have been involved in a still-ongoing series of citizens’ dialogues on future technologies initiated and led by the German Federal Ministry of Education and Research. At least in the German context, these dialogues are to be valued as a unique experiment. On the one hand, several thousand citizens will be involved in the whole procedure. On the other hand, the strong position of the ministry, which is responsible for the entire process and heavily involved in its planning, organization and communication, constitutes an unusual feature. In the paper, the authors allow some first-hand insights into the political background, associated expectations and practical restrictions those procedural innovations are confronted with. Based on first evaluations and internal reflections on the process, they tentatively conclude that the high efforts to guarantee a kind of statistical representativeness are still contested by participants as well as a variety of incumbent political actors.

The next paper also deals with new methodological directions in the field of pTA. Niklas Gudowsky, Walter Peissl, Mahshid Sotoudeh and Ulrike Bechtold describe a recently developed method that allows for comprehensive participatory forward-looking activities. This method, called CIVISTI, brings together expert, stakeholder and lay knowledge in a well-balanced way, preparing long-term oriented recommendations for decision-making in issues related to science, technology and innovation. It comprises three phases. In an initial phase, the invited citizens produce future visions in a bottom-up process. Experts translate these visions into practical recommendations in a consecutive phase. Finally, the same groups of citizens validate and rank the outcome. The authors not only report on first experiences with this new approach, they also address a number of practical challenges and discuss some options for improvement.

Diego Compagna draws our attention to the problems of translation between design and use in participatory technology development projects. His empirical material stems from a recently finished 3-year project on service robots in elderly care. Using some analytical concepts taken from classical social constructivist approaches and actor-network theory, Compagna unrolls step by step and reflects on experiences made in the project. He addresses scenarios as developed by designers, developers and future users involved as ‘translation tools’ and ‘epistemic objects’ that are able to mediate between diverse expectations and experiences. However, as the process continues, scenarios gain a kind of agency and each participating group is forced to align itself to the scenarios. On a more general level and with regard to similar situations in pTA exercises, Compagna concludes that participatory methods such as scenario exercises must be understood as active translators with the intrinsic ability to recompile and reconfigure the whole process in an unexpected way.

In the final paper, Michael Zschiesche offers the opportunity to reflect on pTA in a similar way by providing insights from a related but quite different field of infrastructure projects. In Germany, formal public participation is required in authorization processes according to the Federal Immission Control Act for the approval of industrial facilities as well as in the planning permission procedure for infrastructure projects. Empirical data on those approval procedures show that the right of the concerned publics to be involved in the procedures is not at all made use of in many cases. In particular, procedures according to the Immission Control Act show extremely low rates of participation. Here, only one out of three authorization processes is met by public engagement. Based on secondary sources, Zschiesche also shows that, even in cases where public participation takes place, the actual influence on the outcome remains marginal. To improve the formalized procedure in the future, the author discusses options to combine formal and informal methods—as widely used in pTA—and calls for participatory interventions at much earlier stages of a planning process.

The various papers, hence, cover a wide range of positions and empirical case studies. They also allow for some tentative conclusions in line with recent scholarly discussion: As long as TA positions itself as a mediator between science, politics and the public, it has to cope with the multiplicity of participatory methods and strategies. In addition, it must be able to master specific qualities and the limitations of pTA as well as being prepared to adapt methods and methodologies to changing socio-political environments (Rask et al. 2012). Public discourses on emerging technologies and their possible consequences for society and the environment need not be restricted to policy advice as typically provided by TA institutions. Forms of civic expertise with a special focus on societal impacts may play a stronger role both in technology policy (Stirling 2008) and in technology design (Stewart and Hyysalo 2008). TA may profit from such outreach as these other fields may profit from the procedural and methodological expertise TA has developed during the last 30 years. The papers in this special issue once more contribute to this stock of knowledge and clearly offer some fruitful ideas about promising future directions of pTA theory and practice.

## Footnotes

1 TA′11 on ‘Partizipation in Technikfragen—Legitime Hoffnung oder bloße Illusion?’ organized by the Institute of Technology Assessment, Vienna, 20 June 2011.
